# Anti-Inflammatory Drugs for Alcoholic Liver Disease: A Systematic Review on Survival and Adverse Events

**DOI:** 10.1155/ijh/8535952

**Published:** 2025-10-29

**Authors:** Heni Sukma Zulfatim, Visky Afrina, Annette d'Arqom, Quinamora Estevan Sutantyo, Kamolporn Amornsupak, Tiwaporn Nualkaew

**Affiliations:** ^1^Master Program of Basic Medical Sciences, Faculty of Medicine, Universitas Airlangga, Surabaya, Indonesia; ^2^Research Centre in Advancing Community Healthcare-REACH, Universitas Airlangga, Surabaya, Indonesia; ^3^Department of Anatomy, Histology, and Pharmacology, Faculty of Medicine, Universitas Airlangga, Surabaya, Indonesia; ^4^Translational Medicine and Therapeutics Research Group, Universitas Airlangga, Surabaya, Indonesia; ^5^Faculty of Medicine, Universitas Airlangga, Surabaya, Indonesia; ^6^Department of Transfusion Medicine and Clinical Microbiology, Faculty of Allied Health Sciences, Chulalongkorn University, Bangkok, Thailand; ^7^Immunomodulation of Natural Products Research Unit, Chulalongkorn University, Bangkok, Thailand; ^8^School of Allied Health Sciences, Walailak University, Nakhon Si Thammarat, Thailand; ^9^Hematology and Transfusion Science Research Center, Walailak University, Nakhon Si Thammarat, Thailand

**Keywords:** adverse events, alcoholic liver disease, anti-inflammatory, survival

## Abstract

**Aim:**

Alcoholic liver disease (ALD) is a major global health burden, with alcoholic hepatitis (AH) and severe alcoholic hepatitis (SAH) contributing significantly to mortality. Inflammation plays a central role in disease progression, and various anti-inflammatory therapies, particularly corticosteroids, have been employed to improve survival. However, clinical outcomes across different treatments vary. This systematic review is aimed at evaluating the effectiveness of anti-inflammatory pharmacological therapies compared to corticosteroids in improving short-term survival at 1, 3, and 6 months and to assess the incidence of adverse events in patients with ALD.

**Methods:**

The review followed PRISMA guidelines. A comprehensive literature search was conducted in PubMed, Scopus, ScienceDirect, and Clarivate Web of Science using MeSH terms. Inclusion criteria consisted of full-text, open-access, English articles (2014–2024) that reported survival outcomes and adverse events in patients with ALD treated with corticosteroids versus alternative or adjunctive anti-inflammatory therapies. Studies lacking a corticosteroid comparator were excluded.

**Results:**

Nine randomized controlled trials (RCTs) involving patients with AH and SAH were included. The interventions compared to corticosteroids included pentoxifylline, anakinra, metadoxine, S-adenosylmethionine (SAMe), granulocyte colony-stimulating factor (G-CSF), rifaximin, and fecal microbiota transplantation (FMT) as monotherapies or combination regimens. Among anti-inflammatory therapies, combination therapy with corticosteroids and metadoxine significantly improves 3- and 6-month survival rates in patients with ALD. Similarly, corticosteroids combined with SAMe demonstrate efficacy in enhancing 1- and 6-month survival rates. Notably, the metadoxine-based combination regimen exhibited a superior safety profile, with fewer adverse events compared to other anti-inflammatory therapies evaluated in this review.

**Conclusions:**

Even though corticosteroids remain the current standard of care for severe AH, this review suggests that certain combination therapies, particularly those involving metadoxine or SAMe, may offer some survival benefits. FMT also shows promise by potentially improving survival while maintaining a favorable safety profile. Among these, the metadoxine-based regimen has been explored as a promising therapeutic strategy in some contexts. However, these findings must be interpreted with caution. The evidence is limited by significant study heterogeneity and a lack of high-quality RCTs. These limitations underscore the critical need for well-powered, rigorous RCTs with standardized survival and safety outcomes.

## 1. Introduction

Alcoholic liver disease (ALD) is a major global health concern due to chronic and excessive alcohol consumption. It remains a leading cause of morbidity and mortality worldwide, with increasing incidence in regions of high alcohol consumption. In 2019, alcohol use contributed to approximately 2.6 million deaths, accounting for 4.7% of global mortality. The highest alcohol consumption was in Europe (9.2 L) and America (7.5 L). Rising alcohol consumption trends have been observed in parts of Asia, including India, Japan, China, and Africa. Globally, over two billion individuals consume alcohol, with more than 75 million diagnosed with alcohol use disorders, placing them at increased risk of developing alcohol-related liver diseases [1–3].

**Figure 1:**
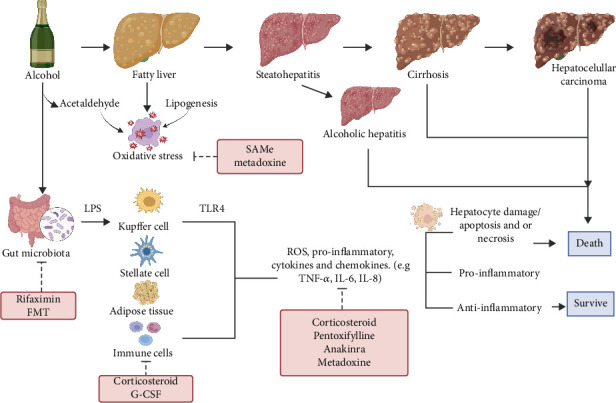
Pathophysiology of ALD and mechanisms of action of key therapeutics. This figure illustrates the progression of ALD driven by alcohol-induced oxidative stress and gut dysbiosis, activating LPS–TLR4 signaling and proinflammatory cytokines (e.g., TNF-*α*, IL-6, and IL-8), leading to hepatocyte injury. Therapeutics such as corticosteroids, pentoxifylline, SAMe, metadoxine, anakinra, G-CSF, and gut-targeted agents (rifaximin and FMT) are aimed at suppressing inflammation, modulating immune responses, and improving clinical outcomes.

ALD represents a pathological spectrum of liver damage, from hepatic steatosis to alcoholic hepatitis (AH), fibrosis, cirrhosis, and hepatocellular carcinoma, indicating progressive severity [4, 5]. Hepatic steatosis, the earliest and most prevalent manifestation, affects over 90% of individuals consuming four to five standard drinks daily [6]. AH, characterized by severe inflammation, is associated with significant short-term mortality, emphasizing the need for early screening and intervention [7, 8]. Key pathogenic mechanisms include oxidative stress induced by reactive oxygen species (ROS), immune dysregulation, and gut–liver axis disruption. Alcohol-induced intestinal permeability facilitates bacterial toxin translocation to the liver, triggering inflammatory cascades and inflammasome activation, wherein stress-induced protein complexes drive inflammatory cytokine production [9]. Proinflammatory mediators, including cytokines such as TNF-*α*, IL-1*β*, IL-6, chemokines, and various immune cells, play pivotal roles in ALD pathogenesis (Figure 1) [10].

Despite increased understanding of ALD pathophysiology, its management remains a formidable challenge. While sustained alcohol abstinence is fundamental, it often proves insufficient in halting disease progression, particularly in patients with advanced liver damage. Furthermore, current pharmacological interventions are limited by suboptimal efficacy and adverse event profiles [11, 12]. Current guidelines recommend corticosteroids as primary treatment for AH in patients with poor prognostic scores and pentoxifylline as a secondary option in select cases [10]. Emerging research has identified novel therapeutic strategies targeting inflammatory pathways, oxidative stress, and liver regeneration [13]. These include TNF-*α* receptor antagonists, antioxidant agents, gut microbiota modulation, and stem cell–based therapies [13, 14]. Although promising, their efficacy and safety require further validation through clinical trials. Studies suggest specific pharmacological agents including corticosteroids (e.g., prednisolone), S-adenosylmethionine (SAMe), interleukin-1 (IL-1) receptor antagonists (e.g., anakinra), metadoxine, rifaximin, and fecal microbiota transplantation (FMT) may improve survival outcomes in ALD patients (Figure 1) [15–17].

Despite numerous studies evaluating individual pharmacological interventions for ALD, a comprehensive comparison between corticosteroids, the current standard of care, and other emerging anti-inflammatory agents remains lacking. Prior reviews have either focused narrowly on single agents (e.g., pentoxifylline) or failed to integrate survival outcomes alongside adverse event profiles. In recent years, various anti-inflammatory agents have been investigated as potential therapies for ALD due to their ability to modulate key pathogenic mechanisms such as inflammation, oxidative stress, and gut–liver axis disruption. These agents include pentoxifylline, anakinra, metadoxine, SAMe, G-CSF, rifaximin, and FMT. Each of these therapies offers unique mechanisms of action and potential benefits.

Pentoxifylline is a nonselective phosphodiesterase inhibitor; it exerts anti-inflammatory effects by increasing intracellular cyclic adenosine monophosphate (cAMP) levels and downregulating TNF-*α* production, thereby attenuating liver inflammation and necrosis [18]. Anakinra, as an IL-1 receptor antagonist, reduces hepatic inflammation by blocking IL-1*β* signaling and downstream inflammasome activation [19, 20]. Metadoxine, a synthetic antioxidant compound, works by accelerating ethanol metabolism, reduces oxidative stress, and decreases proinflammatory cytokines such as TNF-*α* [21]. SAMe has a role as a methyl donor that enhances glutathione synthesis and exerts antioxidant and antifibrotic effects [22]. G-CSF, which stimulates the mobilization of hematopoietic stem cells, promotes liver regeneration through improved neutrophil function [23]. Rifaximin, a gut-selective antibiotic, modulates intestinal microbiota composition, reduces endotoxemia, and downregulates toll-like Receptor 4 (TLR4)-mediated liver inflammation [24]. FMT restores microbial diversity and homeostasis in the gut–liver axis, thereby reducing systemic inflammation [25].  Table 1 summarizes the anti-inflammatory therapies investigated for ALD.

This systematic review addresses these gaps by integrating recent quantitative evidence comparing corticosteroids with a range of anti-inflammatory therapies including anakinra, metadoxine, SAMe, G-CSF, rifaximin, and FMT in patients with AH and SAH. Uniquely, this review evaluates both short-term survival 1-, 3-, and 6-month outcomes and adverse events across interventions, providing an up-to-date, comparative overview to inform clinical decision-making and future therapeutic development.

## 2. Materials and Methods

### 2.1. Study Design

This systematic review was conducted following a structured approach, including the formulation of the research question, definition of eligibility criteria, comprehensive literature search, screening and selection of studies, quality appraisal, and data extraction and synthesis [27]. The review adhered to the PRISMA guidelines. Using the PICO framework, this review focused on adult patients diagnosed with ALD, including those with AH or alcoholic cirrhosis. The intervention of interest comprised anti-inflammatory pharmacological therapies, administered either as monotherapy or in combination with corticosteroids. The comparator group received corticosteroid therapy alone or in combination with placebo. The primary outcome was short-term survival at 1, 3, and 6 months, while the secondary outcome was the incidence of adverse events associated with the interventions. This framework allowed for a systematic and comprehensive evaluation of the efficacy and safety of anti-inflammatory therapies in ALD management.

Studies were eligible if they met the following criteria: RCTs, cohort studies, and case–control studies focused on ALD, published between 2014 and 2024 with full-text availability in English. Included studies were required to report survival outcomes or adverse events. Exclusion criteria were as follows: studies using a comparator group treated with agents other than corticosteroids, studies involving non-ALD populations, and studies with incomplete or irrelevant data.

### 2.2. Search Strategy

A comprehensive literature search was performed across four electronic databases, including PubMed, Scopus, ScienceDirect, and Clarivate Web of Science. The following search terms and Boolean operators were used: (Alcoholic Liver Disease) OR (alcoholic hepatitis) OR (alcoholic stetohepatitis) NOT (Non-Alcoholic) AND (Glucocorticoid) OR (Pentoxifylline) OR (N-acetylcysteine) OR (Granulocyte Colony-Stimulating Factor) OR (interleukin 1 Receptor Antagonist Protein) OR (metadoxine) OR (Rifaximin) OR (Fecal Microbiota Transplantation).

### 2.3. Quality Assessment

The methodological quality of the included studies was assessed using the Risk of Bias 2 (RoB 2, Cochrane 22), which is specifically designed for RCTs. Two independent reviewers evaluated each study across five domains: bias arising from the randomization process, bias due to deviations from intended interventions, bias due to missing outcome data, bias in measurement of the outcomes, and bias in selection of the reported result. Disagreements between reviewers were resolved through discussion or consultation with a third reviewer.

### 2.4. Data Extraction

Data were extracted using a standardized form capturing the following information such as the study objective, population characteristics, study design, setting, pharmacological interventions, follow-up duration, number of participants, age, gender, MELD (Model for End-Stage Liver Disease) score, and MDF (Maddrey discriminant function) score. An MDF score ≥ 32 indicates severe AH requiring treatment initiation, while a MELD score ≥ 20 predicts a high 90-day mortality risk [28, 29].

Additional data extracted included survival outcomes, adverse events, and risk of bias. The control group consisted of participants receiving corticosteroids, either as monotherapy or in combination with placebo. Survival was assessed at 1, 3, and 6 months and was reported using Kaplan–Meier survival curves, noninferiority testing, Lille scores, and log-rank tests. Adverse events were defined as any undesirable medical occurrences and were categorized based on their frequency and severity across treatment arms.

### 2.5. Data Analysi

Due to clinical and methodological heterogeneity among the included studies, a narrative synthesis was conducted. Meta-analysis was not performed owing to variations in study designs, types of interventions, and outcome measurements.

## 3. Result

### 3.1. Search Results and Included Studies

**Figure 2:**
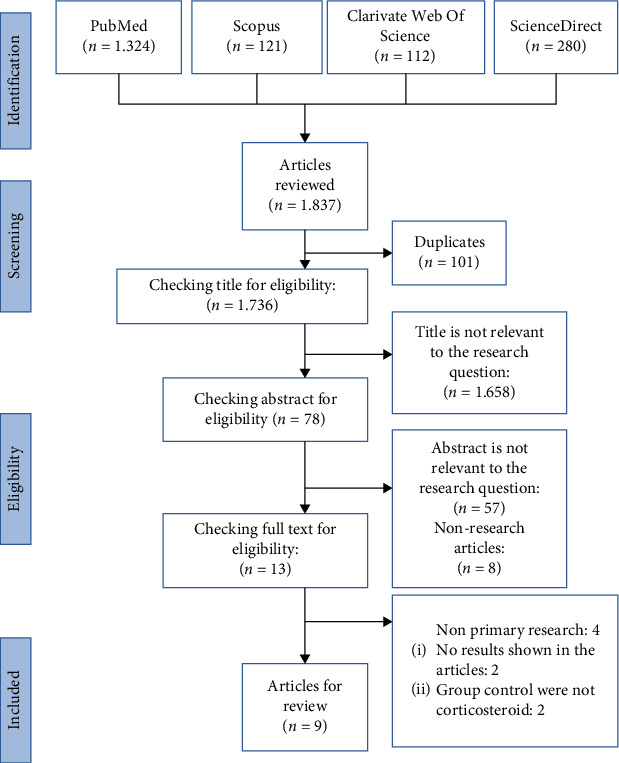
PRISMA flowchart of the literature search process.

The initial search identified 1837 articles across four databases: PubMed (1324), Scopus (121), Clarivate Web of Science (112), and ScienceDirect (280). After removing 101 duplicates, 1736 articles remained for title screening. Of these, 1658 were excluded due to irrelevance to the research question. The abstracts of the remaining 78 articles were assessed, and 65 were excluded (57 unrelated to the topic and 8 nonresearch articles). Thirteen full-text articles were reviewed for eligibility, and four were excluded: Four were nonprimary studies (*n* = 4), two showed no results, and two used control groups that did not receive corticosteroids. Ultimately, nine articles met the inclusion criteria and were included in the systematic review (Figure 2).

### 3.2. Characteristics of Included Studies

All selected articles were RCTs [18–26]. Four trials were conducted in the Region of Americas (United States and Mexico) [19–21, 23], two in Western Pacific Region (Korea) [18, 24], two in European Region (Russia and United Kingdom) [22, 26], and one in the Southeast Asia Region (India) [25]. Seven trials focused on SAH [18–22, 24, 25], while two addressed AH [23, 26]. Interventions included pentoxifylline, G-CSF, anakinra, metadoxine, SAMe, rifaximin, and FMT with standard medical therapy for both treatment and control groups. Article characteristics are in Table 2.

The nine studies included 1803 participants. Three trials with 1309 participants compared corticosteroid with pentoxifylline [18, 21, 26]. Two trials with 250 participants compared corticosteroid with anakinra (alone or with pentoxifylline) and zinc [19, 20]. One trial with 135 participants assessed corticosteroid versus metadoxine [21], another with 34 participants evaluated corticosteroid against G-CSF [23], and the final trial with 40 participants compared corticosteroid with corticosteroid and SAMe [22]; another with 50 participants compared corticosteroid or pentoxifylline alone versus combination with rifaximin [24], and one study with 120 participants compared corticosteroids with FMT [25]. Among participants, 716 had SAH, and 1087 had AH. Mean age ranged from 42.4 to 50.4 years, with more males (1239 males vs. 564 females). Disease severity had a MELD score from 21.2 to 31 and a MDF score from 22.4 to 93.4, reflecting liver dysfunction severity.

### 3.3. Risk of Bias and Quality Assessment

**Figure 3:**
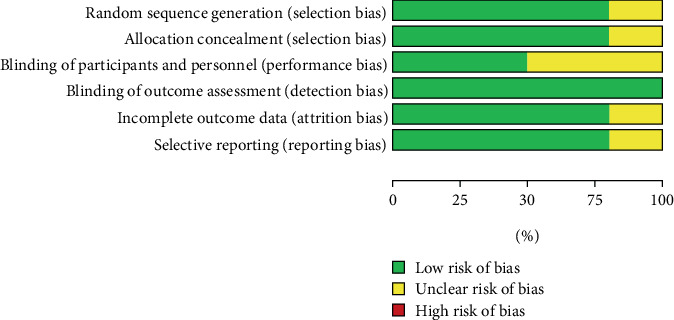
Risk of bias graph.

**Figure 4:**
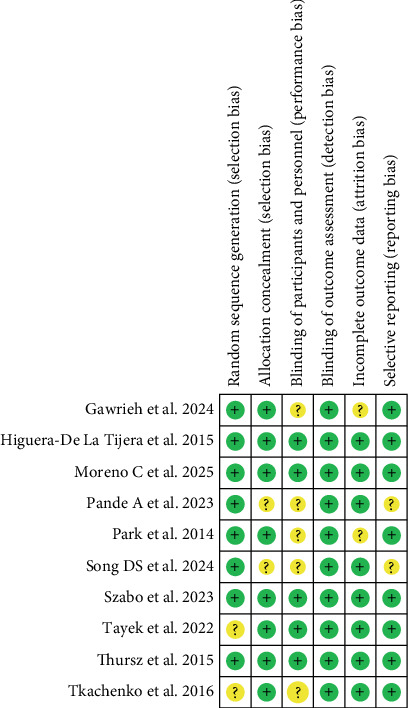
Risk of bias summary.

The risk of bias assessment for included studies used the RoB 2.0 tool for RCTs. Among the nine RCTs, two had a moderate risk of bias in Domain 1 (randomization process), while the remaining six had a low risk across all domains. No trials were excluded, as all were methodologically relevant and provided consistent results (Figures 3 and 4).

### 3.4. Survival and Adverse Events

The effectiveness of anti-inflammatory drugs was evaluated by analyzing survival rates at 1, 3, and 6 months (Table 3). Adverse events were also assessed to determine drug safety (Table 4).

#### 3.4.1. Corticosteroid Versus Pentoxifylline

Three trials compared survival between corticosteroid and pentoxifylline, with two focusing on SAH [18, 21] and one in AH patients [26]. Two trials assessed 28-day survival; corticosteroid showed a slightly higher rate (88.1%) than pentoxifylline (85.7%), with 280 of 325 versus 255 of 320 survivors, respectively [18, 26]. Two trials evaluated 90-day outcomes; survival was higher with pentoxifylline (68.1%, 119/271 participants), though the difference was not statistically significant; corticosteroid had 168 of 276 survivors versus 171 of 268 with pentoxifylline [21, 26]. At 180-day survival, corticosteroid showed better survival in two studies (72.9% and 20%) [18, 21], while one reported higher survival with pentoxifylline (43.9%, 119/271 participants) [26]. Overall, corticosteroid slightly outperformed pentoxifylline across 1–6 months, although outcomes varied by dose and patient characteristics. One trial compared corticosteroid with combination therapy corticosteroid plus pentoxifylline [26]. One-month survival rates were similar (86.7% vs. 86.5%), but combination therapy showed higher survival at 3 (73.6% vs. 66.8%) and 6 months (50.4% vs. 42.3%).

Adverse events were variably reported; Park et al. (2014) noted that hepatorenal syndrome and infections were more frequent with corticosteroid, while pentoxifylline was associated with more gastrointestinal bleeding (GIB) [18]. Thursz et al. (2015) found infections were most common with corticosteroid (24%), while gastrointestinal and hepatobiliary disorders were higher with pentoxifylline [26]. Another trial also reported higher hepatic encephalopathy and hepatorenal syndrome in both groups, with slightly higher rates in the corticosteroid group [21]. Overall, gastrointestinal and hepatobiliary events were more frequent with pentoxifylline, while infections, hepatorenal syndrome, and encephalopathy were more common with corticosteroids. Combination therapy was associated with the lowest incidence of adverse events.

#### 3.4.2. Corticosteroid Versus Anakinra

Two trials compared corticosteroid and anakinra in SAH patients, both using methylprednisolone in the corticosteroid group [19, 20]. In the anakinra group, one trial used anakinra with zinc, while the other combined it with pentoxifylline and zinc. A total of 123 participants received corticosteroids, and 127 received anakinra. In the study by Gawrieh et al., 1-, 3-, and 6-month survival was higher in the corticosteroid group (97%, 90%, and 81%) [20], while Szabo et al. reported slightly better survival in the anakinra combination group (86.8%, 69.8%, and 67.9%) [19]. However, no significant differences were foutablnd between groups. Overall, 1-month survival was 91.06% (corticosteroid) versus 85.8% (anakinra), 3-month survival was 77.2% versus 72.4%, and 6-month survival was 70.7% versus 67.7%, favoring corticosteroids slightly across all time points.

Adverse events were more common in the anakinra group, including higher rates of acute kidney injury (AKI) (44.6%), infections (31.1%), and serious adverse events (66.2%) [19, 20]. The limitation was suboptimal monitoring due to the COVID-19 pandemic [20]. Szabo et al. found that renal and urinary disorders (30%), fungal infections (10%), vascular disorders (22%), and GIB (8%) were more frequent in the corticosteroid group, while hepatobiliary disorders were more common in the anakinra combination group [19].

#### 3.4.3. Corticosteroid Versus Combination (Corticosteroid and Metadoxine)

One trial compared corticosteroid (prednisone) versus corticosteroid combined with metadoxine in SAH patients [21]. Although 1-month survival was not reported, 3- and 6-month survival rates were higher in the combination group (68.6% and 48.6%) compared to corticosteroid alone (both 20%), suggesting that metadoxine improved survival outcomes in SAH. Higuera et al. (2015) found adverse events were more frequent in the corticosteroid group, including hepatic encephalopathy (60% vs. 28.6%), hepatorenal syndrome (54.3%), variceal bleeding (37.1%), and infections (40%). Other adverse events comprised urinary tract infections, spontaneous bacterial peritonitis, pneumonia, esophageal candidiasis, and diarrhea. Metadoxine also showed benefit in reducing alcohol relapse, a key factor in 6-month mortality [21].

#### 3.4.4. Corticosteroid Versus Combination (Corticosteroid and SAMe)

One trial investigated prednisolone versus a combination of prednisolone and SAMe in SAH patients [22]. At 1 month, survival was 100% combination group and 90% in the combination group (90%) compared to corticosteroid alone (75%). Although the difference was not statistically significant, the Lille model indicated improved survival with the combination (95% vs. 65%, *p* = 0.044). Tkachenko et al. (2016) reported adverse events including hepatorenal syndrome (20% in the corticosteroid group, none in combination), infections (25% vs. 30%), pneumonia (nosocomial; two in corticosteroid group and bronchial; four in combination group), and hyperglycemia (35% vs. 30%) [22].

#### 3.4.5. Corticosteroid Versus Combination (Corticosteroid and G-CSF)

One trial compared corticosteroid monotherapy (prednisolone 40 mg/day) with a combination of prednisolone and G-CSF (pegfilgrastim 0.6 mg subcutaneously) in AH patients [23]. Due to FDA safety limits, pegfilgrastim was administered only twice within 2 weeks. At 1, 3, and 6 months, survival was consistently higher in the corticosteroid group (94.4%, 83.3%, and 72.2%) compared to the combination group (75.0%, 68.8%, and 68.8%), but without statistical significance (*p* > 0.05%). Tayek et al. (2022) reported adverse events including AKI, hepatorenal syndrome, and infections. AKI increased in the corticosteroid group (from two to three cases), while the combination group remained at four cases. Hepatorenal syndrome occurred in two participants per group. Infections were slightly more common in the corticosteroid group (five vs. four cases by Week 12). Most deaths were due to liver failure, without identifiable precipitating factors [23].

#### 3.4.6. Corticosteroid Versus Combination (Corticosteroid and Rifaximin)

One trial assessed the addition of rifaximin to corticosteroid or pentoxifylline in patients with SAH. A total of 50 patients were enrolled (29 in control and 21 in rifaximin group). At 90 and 180 days, liver transplant (LT)–free survival did not differ significantly between the control and rifaximin group (86.2% vs. 73.6%, *p* = 0.289 at 90 days, and 68.3% vs. 61.3%, *p* = 0.502 at 180 days). When stratified by treatment, rifaximin did not improve survival in either corticosteroid (*p* = 0.186) or pentoxifylline groups (*p* = 0.548). Adverse events, including liver-related complications, occurred at similar rates in both groups, and rifaximin showed no clear preventive effect [24].

#### 3.4.7. Corticosteroid Versus FMT

One trial compared FMT with prednisolone (40 mg/day for 28 days) in 120 SAH patients. At 90 days, survival was significantly higher in the FMT group than in the prednisolone group (75.0% vs. 56.6%, *p* = 0.044). Although 28-day survival showed no significant difference (88.3% vs. 78.3%, *p* = 0.243), FMT was associated with fewer infection-related deaths (3.6% vs. 19.3%, *p* = 0.01). Microbiome analysis showed a favorable shift toward beneficial taxa and improved microbial diversity by Day 28. Common adverse events in the FMT group were mild, such as bloating and nasopharyngeal [25].

## 4. Discussion

AH, particularly in its severe form (SAH), is associated with high short-term mortality. Controversy persists regarding the effectiveness of available therapies for AH. Treatments include corticosteroid, pentoxifylline, metadoxine, and G-CSF [30, 31]. In addition, recent studies have explored the potential of emerging therapies such as rifaximin and FMT which target gut–liver axis modulation, microbiome restoration, and hepatocellular protection, respectively. Despite the availability of several anti-inflammatory therapies, clinical outcomes remain inconsistent, and adverse events are a major concern. To our knowledge, this is the first systematic review to directly compare combination anti-inflammatory therapies to corticosteroids in patients with AH and SAH, with a focused evaluation of both survival outcomes and adverse events across short-term intervals of 1, 3, and 6 months.  Table 1 summarizes the mechanism of action, recommended doses, and adverse events of anti-inflammatory therapies investigated for ALD.

Corticosteroids are steroid hormones produced by the adrenal glands in response to ACTH and regulated by hypothalamic CRH. Common corticosteroids include prednisolone, prednisone, and methylprednisolone, used to treat various conditions including AH [32]. Prednisolone is recommended by AASLD and EASL as the first-line treatment for AH and SAH. The standard regimen is 40 mg of prednisolone or 32 mg of methylprednisolone daily for 28 days, followed by tapering over 3 weeks. It is prescribed for patients with MELD scores ≥ 32 or GAHS ≥ 9 and continued if the Lille score is < 0.45 and there are no contraindications [28, 33, 34]. Corticosteroids have shown a 30-day survival benefit in SAH patients, particularly those with MELD scores between 25 and 3938, with a 59% feasibility rate and a 75.4% benefit rate for AH treatment [35]. Studies indicate that corticosteroids improve short-term survival in AH patients [36]. A global cohort study supports the 30-day survival benefit of corticosteroid use in AH, although they do not impact long-term survival [21].

### 4.1. Corticosteroid Versus Pentoxifylline

This review compares the effectiveness and safety of corticosteroids and pentoxifylline in the management of AH. Two trials showed corticosteroids had higher survival rates at 1 and 6 months compared to pentoxifylline, but no difference at 3 months [18, 26]. A meta-analysis found prednisolone reduced mortality at 28 days compared with pentoxifylline [37]. Previous studies indicated pentoxifylline is less effective than corticosteroids for improving survival in SAH patients [38]. Despite its inferior efficacy, pentoxifylline remains a potential alternative when corticosteroids are contraindicated, such as in cases of active infections, AKI, and GIB [33, 39, 40]. When combined with corticosteroids, pentoxifylline did not significantly improve survival, although the corticosteroid group showed higher survival at 1 month and the combination group at 3 and 6 months. These findings are consistent with previous studies [38, 40]. As pentoxifylline showed no significant survival benefit, it is no longer recommended for AH treatment [28, 33].

Corticosteroids, particularly prednisolone, continue to serve as the first-line treatment due to their proven short-term survival advantage. Nevertheless, their immunosuppressive effects increase infection risk [35]. Pentoxifylline, an anti-inflammatory phosphodiesterase inhibitor, increases cAMP, has antioxidant properties, and reduces inflammation and hepatorenal syndrome incidence, but does not improve short-term survival. Combined, corticosteroids and pentoxifylline can lower infection and hepatorenal syndrome risks [30, 36, 41].

In terms of safety, both therapies exhibit comparable adverse event profiles. Infections were more frequent with corticosteroids, while GIB was more common in the pentoxifylline group. No significant difference in the overall incidence of adverse events was observed between the two treatments [18, 21, 26].

### 4.2. Corticosteroid Versus Anakinra

Anakinra, an IL-1 receptor antagonist, has shown potential in reducing inflammation and liver damage in ALD by targeting proinflammatory pathways [42]. Originally used in acute and chronic inflammatory conditions [43], it is now being explored as an alternative for patients unresponsive to corticosteroids [44]. In this review, corticosteroids demonstrated superior survival outcomes at 1 and 6 months compared to anakinra. However, at 3 months, the combination of anakinra and pentoxifylline yielded a competitive survival rate (67.9%) compared to corticosteroids (81%), suggesting a potential benefit in selected cases. Although corticosteroids remain the first-line therapy due to their effectiveness, anakinra either as monotherapy or in combination may serve as a promising option, particularly in patients unsuitable for corticosteroid therapy. Nevertheless, further clinical trials are needed to validate its efficacy and define optimal treatment strategies. Individualized approaches based on patient profiles are essential to improve clinical outcomes.

Regarding safety, anakinra is generally well-tolerated with mild side effects and minimal liver injury [45]. However, this review found a higher incidence of SAEs in the anakinra combination group compared to corticosteroid, although the difference was not substantial. Both treatments require vigilant monitoring, particularly in high-risk patients. Both anakinra and corticosteroids showed similar rates of infections, which occurred exclusively in the corticosteroid group (five cases) [19], likely due to their stronger immunosuppressive effects [46]. Thus, anakinra may offer a safer profile concerning opportunistic infections, especially in immunocompromised individuals [19]. Preclinical studies have shown that anakinra can reduce liver inflammation and neutrophil infiltration in animal models [47], as well as attenuate lipopolysaccharide (LPS)-induced renal and hepatic injury [48]. Inhibition of NLRP3 inflammasome activation and IL-1*β* has been shown to reduce alcohol consumption [49]. However, an increased incidence of AKI was observed in patients receiving anakinra combinations compared to corticosteroids [20]. In the trial, the difference in AKI occurrence between groups was small, suggesting AKI risk may vary depending on therapy combination and context [19]. Anakinra is associated with a higher incidence of AKI than corticosteroid, requiring closer monitoring of kidney function. While anakinra offers benefits in managing inflammation, the risk of side effects, particularly AKI and other SAEs, is higher with the anakinra combination. Therapy choices should consider the patient's risk profile with careful monitoring of side effects, especially in patients at high risk for kidney complications or infections.

### 4.3. Corticosteroid Versus Combination (Corticosteroid and Metadoxine)

In addition, another medication compared to corticosteroid in this review was metadoxine. The combination of corticosteroid and metadoxine may increase short-term survival in AH [21]. Metadoxine as an adjunct therapy can protect the liver. Metadoxine is a synthetic antioxidant compound (pyridoxine and pyrrolidone carboxylate) with strong antioxidant properties, beneficial for preventing early stage liver damage by avoiding redox imbalance in hepatocytes and reducing TNF-*α*, a key event in liver injury initiation [48, 50]. Previous reviews indicated that metadoxine can effectively treat alcohol-related issues, owing to its ability to metabolize toxicity and promote rapid clinical recovery [51]. In patients with alcoholic fatty liver disease, metadoxine treatment resulted in significant improvement in liver function tests, with normalization at treatment end [52]. This review provides strong evidence that metadoxine may be a more effective therapeutic option than corticosteroid for improving survival rates in SAH patients, both short and long terms.

The trial review results showed that adverse events like hepatic encephalopathy and hepatorenal syndrome were less frequent in the combination group. There were no significant differences in variceal bleeding or infection incidence between the combination and prednisolone groups. Metadoxine may help prevent alcohol relapse [21]. These findings suggest that metadoxine is well-tolerated by patients undergoing treatment for alcohol-related liver disease. Previous studies report that metadoxine can effectively improve liver steatosis, though it does not significantly affect liver histology or ALT/AST levels compared to placebo in NASH patients [53]. Metadoxine can reduce alcohol's toxic effects and prevent ALD progression by reducing liver inflammation through inhibiting immune cell infiltration, evidenced by reduced ALT, AST, ALP, TNF-*α*, IL-6, and NLRP3 levels in liver tissue, along with decreased liver enzymes, improved AST/ALT ratio, neutralized free radicals, and increased glutathione levels [54, 55]. Metadoxine administration has a favorable pharmacokinetic profile and minimal side effects [56]. Metadoxine may be a promising therapy for ALD management, potentially reducing inflammation, improving liver function, and reducing alcohol relapse.

Overall, despite the higher frequency of side effects in the corticosteroid group, this study supports using a combination of metadoxine as a more effective therapy for improving survival of patients with SAH. Metadoxine administration shows additional benefits in reducing the risk of alcohol relapse, a key factor influencing the prognosis of SAH patients. Therefore, metadoxine may be considered a safer and more effective alternative to corticosteroid for managing SAH patients. Unfortunately, despite these promising results, metadoxine has not been investigated in large-scale studies.

### 4.4. Corticosteroid Versus Combination (Corticosteroid and SAMe)

This review reports that combining corticosteroid and SAMe resulted in higher survival rates at 1 and 6 months, but no significant difference was found between the groups [22]. This suggests that adding SAMe to corticosteroid treatment for AH does not significantly improve survival. SAMe can reduce liver fibrosis and damage in mouse models due to its antioxidant role, reducing oxidative stress, and its anti-inflammatory properties through inhibiting signaling pathways like NF-*κ*B [57–60]. Clinical trials in humans have yielded varying results. One study found no significant effect of SAMe on overall mortality or liver-related deaths in ALD patients [60]. SAMe also enhances CD4+ T-cell survival, positively affecting systemic survival, especially in individuals with immune dysfunction due to alcohol abuse [59]. Recent reviews report SAMe improves liver health parameters [61]. However, earlier reviews suggest SAMe benefits liver function in ALD patients, but it does not significantly affect mortality rates [60].

The combination group of prednisolone and SAMe included infections, nosocomial pneumonia, bronchial pneumonia, spontaneous bacterial peritonitis, and hyperglycemia [22]. This indicates that SAMe, as an adjunct treatment, can reduce side effects in the AH. Previous reviews reported no significant association between SAMe and mild adverse effects, with no serious events in ALD patients [60]. A recent review also reported that SAMe causes minimal side effects, generally mild gastrointestinal disturbances [61. A positive finding was the absence of hepatorenal syndrome in the combination group, while it was observed in 20% of patients in the prednisolone group [22]. This suggests that SAMe may reduce hepatorenal syndrome events. SAMe exhibits nephroprotective effects by stabilizing cell membranes, reducing cytolysis, and improving microcirculation, which helps prevent kidney fibrosis and preserve renal function. SAMe enhances albumin synthesis in the liver and improves glomerular filtration rate, which is crucial for kidney health [62–64]. SAMe improves liver function by enhancing mitochondrial fatty acid oxidation and reducing lipid accumulation, beneficial for managing fatty liver disease [65].

### 4.5. Corticosteroid Versus Combination (Corticosteroid and G-CSF)

This review found that combining corticosteroid with G-CSF resulted in lower survival rates at 1, 3, and 6 months compared to corticosteroid alone in SAH and cirrhosis patients [66–69]. Death is generally attributed to liver failure, with no specific cause identified, particularly during the COVID-19 pandemic. A European study reported increased mortality with G-CSF administration, contrary to Asian findings [70], due to G-CSF exacerbating inflammation via TLRs and increasing liver damage, oxidative stress, and inflammasome activation [71, 72]. Although the corticosteroid group showed higher survival rates, results were not conclusive enough to suggest the combination of anakinra (prednisolone and pegfilgrastim) was superior or significantly beneficial.

A key feature of AH and SAH is massive neutrophil infiltration into the liver, which plays a central role in driving inflammation and tissue damage. A recent study demonstrated that sustained IL-8^+^ neutrophils are critical mediators of SAH progression [73]. This raises an important paradox: While G-CSF and corticosteroids are designed to promote immune modulation and regeneration, both treatments also elevate circulating neutrophils. Indeed, in the G-CSF trial [23], marked elevation of neutrophils was observed in SAH patients, which contributed to the FDA's decision to stop the trial due to safety concerns. Thus, although G-CSF can mobilize CD34^+^ stem cells, enhance neutrophil phagocytosis, and support liver regeneration [71–75], its neutrophil-elevating effect may worsen the inflammatory cascade in SAH, potentially explaining the inconsistent survival outcomes across different trials. With regard to renal function, G-CSF showed benefits in treating AKI, improving creatinine levels without significant side effects [75]. In this review, AKI incidence was higher in the combination group than the prednisolone group, but additional AKI events were rare. Interestingly, the prednisolone group experienced more infections than the combination group, suggesting G-CSF might reduce infection risk and enhance steroid response. No hepatic encephalopathy or variceal bleeding occurred, and overall adverse events were comparable between groups, indicating similar safety profiles [23]. Taken together, the role of G-CSF in SAH remains controversial. While it may promote regeneration, its propensity to increase circulating neutrophils, a hallmark of disease progression, raises significant concerns. Future clinical trials should carefully consider the dual effect of G-CSF and corticosteroids on neutrophil biology, as targeting neutrophil-mediated inflammation may provide better therapeutic guidance in SAH. Despite its ability to compare survival and adverse events of several anti-inflammatory drugs for ALD, this study has some limitations, including its absence from a systematic review registry. This absence was due to registry restrictions, which, at the time of this review, limited registrations to COVID-19 studies. Furthermore, meta-analysis was not feasible due to the heterogeneity in study design, population characteristics, interventions, and outcome measures.

### 4.6. Corticosteroid Versus Combination (Corticosteroid and Rifaximin)

Rifaximin has been proposed as an adjunct therapy in SAH due to its ability to modulate the gut microbiota and reduce systemic endotoxemia [76]. It has demonstrated efficacy in reducing complications such as hepatic encephalopathy and hospitalization in cirrhotic patients by decreasing bacterial overgrowth and altering microbial composition, without significant systemic [76, 77]. However, its benefit in the acute inflammatory setting of SAH remains uncertain. A recent randomized controlled trial showed that adding rifaximin to corticosteroid or pentoxifylline therapy did not confer additional survival benefit or prevent liver-related complications in patients with SAH. Specifically, there was no significant difference in 180-day liver transplantation-free survival between the rifaximin and control groups (61.3% vs. 68.3%, *p* = 0.502), and the incidence of complications such as hepatic encephalopathy, infections, and bleeding was comparable [24].

Mechanistically, rifaximin is thought to act by reducing gut-derived LPS and downregulating TLR4 signaling, which are known to exacerbate hepatic inflammation and fibrosis through the gut–liver axis [78, 79]. Nevertheless, in advanced SAH, the extent of microbial translocation and systemic inflammation may exceed the modulatory capacity of rifaximin alone. In such settings, immune dysregulation is often widespread and less responsive to microbiota suppression, particularly when multiorgan failure has already developed [80]. The lack of clinical benefit also raises questions regarding patient selection and timing of therapy [28]. It is possible that rifaximin may exert greater effects in earlier stages of disease or in patients with milder systemic inflammation and preserved intestinal barrier function. Moreover, while rifaximin is well-tolerated and has a favorable safety profile, its limited impact on infection rates or major adverse events in SAH suggests that its microbiome-directed effects may not be sufficient to counteract fulminant inflammatory injury [24].

### 4.7. Corticosteroid Versus Combination (Corticosteroid and FMT)

FMT represents a novel therapeutic strategy in SAH aiming to correct gut dysbiosis and modulate systemic inflammation through restoration of a healthy microbial ecosystem [76]. The gut–liver axis has been increasingly implicated in the pathogenesis of alcohol-associated liver disease, particularly through the role of intestinal permeability, endotoxemia, and immune dysregulation [78]. In this context, FMT has emerged as a promising intervention with both mechanistic and clinical relevance. The randomized trial by Pande et al. (2023) directly compared FMT to prednisolone in patients with SAH and demonstrated that FMT improved 90-day survival and significantly reduced infection-related mortality, one of the leading causes of death in corticosteroid-treated patients. Unlike corticosteroids, which suppress immune function and increase susceptibility to infections, FMT modulates the immune response while preserving host defense mechanisms. The study also reported favorable microbial shifts, including increased diversity and enrichment of beneficial taxa such as Lachnospiraceae, *Prevotella*, and *Veillonella*, suggesting that microbial restoration may be central to FMT's therapeutic effect [25]. These findings are consistent with prior research indicating that FMT can influence systemic immunity, hepatic inflammation, and even alcohol cravings through modulation of microbial metabolites and signaling pathways [81, 82].

In contrast to pharmacologic therapies like rifaximin, which suppress bacterial overgrowth but do not restore microbial diversity, FMT may offer a broader and more durable effect on the gut–liver axis [77]. This is supported by studies showing long-term persistence of donor microbiota posttransplant and associated reductions in inflammatory biomarkers [83]. Clinically, FMT may be particularly beneficial in patients with contraindications to corticosteroids, such as those with concurrent infections, renal dysfunction, or corticosteroid nonresponse [26]. The absence of serious adverse events related to FMT in the trial strengthens its safety profile and potential applicability in routine practice [25]. However, FMT still faces practical and regulatory challenges, including donor selection, standardization of stool preparation, and route of administration [84]. Moreover, long-term outcomes and comparative efficacy across different FMT protocols remain to be fully elucidated. Future research should explore combination strategies integrating FMT with anti-inflammatory or regenerative therapies to target multiple pathways in SAH pathophysiology.

## 5. Conclusion

This review reinforces corticosteroids as the current standard of care in AH, yet it also highlights the unmet need for more effective and safer therapeutic alternatives. Notably, combination corticosteroid with metadoxine or SAMe can improve survival rates in patients. Among these, metadoxine combinations demonstrate a more favorable safety profile with fewer adverse events, though future validation in large-scale RCTs remains a priority. Anakinra and SAMe show promise in selected patient populations, particularly those with corticosteroid contraindications or renal impairment, and their use should be guided by individual risk profiles. FMT emerges as a novel gut-targeted strategy with a favorable safety profile. In contrast, rifaximin has not demonstrated survival benefit, although it may modulate gut–liver inflammation; current evidence remains inconclusive. G-CSF remains highly controversial. Although it may enhance regeneration and reduce infections, its marked induction of circulating neutrophils, a hallmark of severe AH progression, raises significant concerns about long-term safety. The interpretation of these findings is limited by heterogeneity in study designs, treatment regimens, and outcome measures, as well as by inconsistent adverse event reporting and the small number of high-quality trials for several agents. Future research should prioritize well-powered, blinded RCTs evaluating the most promising agents (metadoxine, FMT, and potentially SAMe), with standardized survival and safety endpoints.

## Figures and Tables

**Table 1:** Summary of anti-inflammatory therapies investigated for ALD.

**No.**	**Drug name**	**Mechanism of action**	**Monotherapy/combination**	**Recommended dose**	**Duration**	**Common adverse events**	**Reference**
1	Corticosteroids (e.g., prednisolone)	Suppress proinflammatory cytokines (e.g., TNF-*α* and IL-1*β*), modulate immune response	Monotherapy/combination	40 mg orally once daily	28 days	Infection, hepatorenal syndrome, gastrointestinal bleeding/variceal bleeding, hepatic encephalopathy, renal and urinary disorders	Park et al., [18]; Higuera-De La Tijera et al., [21]; Thursz et al., [26]; Tkachenko et al., [22]; Tayek et al., [23]; Gawrieh et al., [20]; Szabo et al., [19]; Song et al., [24]; Pande et al., [25]
2	Pentoxifylline	Phosphodiesterase inhibitor; increases cAMP and inhibits TNF-*α* production	Monotherapy/combination	400 mg orally three times daily	28–30 days	Gastrointestinal discomfort, hepatobiliary events	Park et al., [18]; Higuera-De La Tijera et al., [21]; Thursz et al., [26]
3	Anakinra	IL-1 receptor antagonist; blocks IL-1*β* signaling and inflammasome activation	Combination (with zinc ± PTX)	Anakinra 100 mg SC daily, zinc 220 mg/day	Anakinra: 14 days Zinc: 90 days	Infection, acute kidney injury, gastrointestinal bleeding, hepatobiliary disorders, SAEs	Gawrieh et al., [20]; Szabo et al., [19]
4	Metadoxine	Antioxidant; enhances ethanol clearance, reduces oxidative stress and TNF-*α*	Combination (with corticosteroids/PTX)	MTD 500 mg TID + PDN or PTX	30 days	Urinary tract infections, spontaneous bacterial peritonitis, pneumonia, esophageal candidiasis, and diarrhea	Higuera-De La Tijera et al., [21]
5	SAMe (S-adenosylmethionine)	Enhances glutathione synthesis, methyl donor, antioxidant, and antifibrotic	Combination (with corticosteroids)	800 mg IV daily + prednisolone 40 mg/day	28 days	Infection, nosocomial pneumonia, hyperglycemia	Tkachenko et al., [22]
6	G-CSF	Stimulates CD34+ stem cells and neutrophil mobilization, promotes liver regeneration	Combination (with corticosteroids)	0.6 mg subcutaneously on Day 1 and Day 8	1–8 days	Infection, hepatorenal syndrome, acute kidney injury	Tayek et al., [23]

**Table 2:** Characteristics of included studies and baseline.

**Author and year**	**Aim**	**ALD**	**Study design**	**Setting**	**Pharmacotherapy**	**Follow-up**	**n**	**Age (SD)**	**Sex (M/F)**	**Bilirubin (mg/dl)**	**Alcohol intake (g/day, SD)**	**MELD**	**Maddrey DF**
Park et al., 2014 [18]	Survival 1 and 6 months	SAH	RCT	Korea	PTX (3 × 400 mg/day) vs. PDN (40 mg/day) Both for 28 days	Weekly in 1 month and bimonthly for 6 months or until death	121	47.8–50.1 (8.6–9.5)	91/30	16.6–18.2	N/A	N/A	64.8–67.7
Higuera-De La Tijera et al., 2015 [21]	Survival 3–6 months	SAH	RCT	Mexico	1. PDN (40 mg/day) 2. PDN (40 mg/day) + MTD (3 × 500 mg/day) 3. PTX (3 × 400 mg/day) 4. PTX (3 × 400 mg/day) + MTD (3 × 500 mg/day) Both for 30 days, or until death if it occurs earlier	Month 1 (weekly), Months 2 and 3 (2×/month), Months 4–6 (monthly)	135	43.1–44.5 (9.0–9.9)	124/11	23.0–25.9	±338.8 ± 167.4	28–31	67.3–93.4
Thursz et al., 2015 [26]	Mortality 28 days	AH	RCT	United Kingdom	1. Placebo/placebo 2. Placebo/PDN (40 mg/day, oral, 28 days) 3. Placebo/PTX (3 × 400 mg/day, oral, 28 days) 4. PTX/PDN	12 months or until the time of their death	1053	48.7 (10.2)	685/407	17.6	N/A	21.2	62.6
Tkachenko et al., 2016 [22]	Survival 28 days	SAH	RCT	Russia	1. PDN 40 mg/day, oral, 28 days 2. PDN + SAMe 800 mg/day, Iv, first 7 days. SAMe 1200 mg/day, oral. For Day 8 until 2 months		40	46.10–46.85	27/13	12.05–13.6	N/A	23.25–23.40	59.9–79.64
Szabo et al., 2023 [19]	Survival 180 days	SAH	RCT	United States	1. Methylprednisolone 32 mg/day (28 days) 2. Anakinra (IL-1RA; 100 mg/day, Cs, 14 days) + PTX (3 × 400 mg/day, oral, 28 days) + zinc (220 mg/day, oral, 180 days)		103	45.3 (10.4)	63/40	19.7	N/A	25.7	59.9
Song et al., 2025 [24]	Survival 3 and 6 months	SAH	RCT	Korea	Rifaximin 400 mg TID + standard therapy (corticosteroids/pentoxifylline) for 4 weeks vs. standard therapy alone	6 months	50	48.4 (9.2)	35/15	19.4	N/A	25.7	73.8
Tayek et al., 2022 [23]	Time to date through 90 days	AH	RCT	United States	1. PDN 40 mg/day or PTX 3 × 400 mg/day, oral, 28 days 2. PDN or PTX + pegfilgrastim 0.6 mg sc on Day 1 and again on Day 8 if the WBC count was < 30,000/mm^3^ on Day 8		34	49.9–50 (12.7–10.9)	34/0	12.8–17.7	N/A	25.6–26.2	53.7–55.2
Gawrieh et al., 2024 [20]	Survival 90 days	SAH	RCT	India	1. Methylprednisolone (40 mg/day, oral, 30 days) 2. Anakinra (100 mg/day, Sc, 14 days) + zinc (220 mg/day, oral, 90 days)		147	43.5–45.2	88/59	17.1–19.4	N/A	24.0–25.0	54.4–56.2
Pande et al., 2023 [25]	Survival 90 days	SAH	RCT	India	FMT vs. prednisolone 40 mg/day for 28 days	28, 90, and 180 days	120	41.98 (8.38)	117/3	18.55	N/A	25.19	66.61

*Note:* The mean and standard deviation were derived from the raw values of the RCT.

Abbreviations: AH, alcoholic hepatitis; F, female; FMT, fecal microbiota transplantation; M, male; Maddrey DF, Maddrey discriminant function; MELD, Model for End-Stage Liver Disease; MTD, metadoxine; PDN, prednisolone; PTX, pentoxifylline; RCT, randomized controlled trial; SAH, severe alcoholic hepatitis; SAMe, S-adenosyl-l-methionine; SD, standard deviation.

**Table 3:** Survival rate of patients with ALD.

**Pharmacology design**		**Author**	**Survival**	**n**	**Control**	**Intervention**	**p** **value**
					**S (%)**	**D/S (%)**	
*CST vs. other anti-inflammatory drugs*							
CST vs. PTX	PDN (monotherapy) vs. PTX	Park et al., [18] SAH (*n* = 121)	1 month	59/62	52 (88.1%)	47 (75.8%)	0.08 noninferiority (Δ15%)
			6 months		43 (72.9%)	40 (64.5%)	0.003 (KM, Lille model)
	PDN vs. PTX	Thursz et al., [26] AH (*n* = 1053)	1 month (28 days)	266/258	228 (85.7%)	208 (80.6%)	0.686
			3 months	241/235	161 (66.8%)	160 (68.1%)	—
			6 months (180 days)	274/271	116 (42.3%)	119 (43.9%)	—
	PDN vs. PTX	Higuera-De La Tijera et al., [21] SAH (n = 135)	3 month	35/33	7 (20%)	11 (33.3%)	—
			6 month		7 (20%)	6 (18.2%)	—
CST vs. anakinra	Methylprednisolone vs. anakinra + zinc	Gawrieh et al., [20] SAH (*n* = 147)	1 month	73/74	71 (97%)	63 (85%)	0.059
			3 months		66 (90%)	55 (70%)	0.018
			6 months		59 (81%)	50 (68%)	0.205
	Methylprednisolone vs. anakinra + PTX + zinc	Szabo et al., [19] SAH (*n* = 103)	1 month (28 days)	50/53	41 (82%)	46 (86.8%)	
			3 months		29 (58.0%)	37 (69.8%)	
			6 months		28 (56.0%)	36 (67.9%)	0.3001
CST vs. FMT	Prednisolone vs. FMT	Pande et al., 2023 [25]	28 days	60/60	47 (78.3%)	53 (88.3%)	0.243
			3 months		34 (56.6%)	45 (75%)	0.044
			6 months		31 (51.7%)	42 (70%)	0.064
*CST vs. combination (CST + others anti-inflammatory drug)*							
CST vs. CST + PTX	PDN + placebo vs. PDN + PTX	Thursz et al., [26] AH (*n* = 1053)	1 month (28 days)	266/260	228 (86.7%)	225 (86.5%)	0.41
			3 months	241/243	161 (66.8%)	179 (73.6%)	—
			6 months	274/272	116 (42.3%)	137 (50.4%)	—
CST vs. CST + MTD	PDN vs. PDN + MTD	Higuera-De La Tijera et al., [21] SAH (*n* = 135)	3 months	35/35	7 (20%)	24 (68.6%)	0.0001 (KM, log-rank test)
			6 months		7 (20%)	17 (48.6%)	0.003 (KM, log-rank test)
CST + SAMe	PDN vs. PDN + SAMe	Tkachenko et al., [22] SAH (*n* = 40)	1 month (28 days)	20/20	18 (90%)	20 (100%)	—
			6 months		15 (75%)	18 (90%)	0.219
CST vs CST + G-CSF	PDN vs. PDN + pegfilgrastim	Tayek et al., [23] AH (*n* = 34)	1 month (31 days)	18/16	17 (94%)	12 (75%)	0.27
			3 months		15 (83.3%)	11 (68.8%)	0.48
			6 months		13 (72.2%)	11 (68.8%)	0.97
CST + rifaximin vs. CST	CST + rifaximin vs. CST	Song et al., [24]	3 months	50	25 (86.2%)	15 (73.6%)	0.289
			6 months	50	20 (68.3%)	13 (61.3%)	0.502

Abbreviations: AH, alcoholic hepatitis; CST, corticosteroid; D/S, death/survive; FMT, fecal microbiota transplantation; G-CSF, granulocyte colony-stimulating factor; MTD, metadoxine; PDN, prednisolone; PTX, pentoxifylline; S, survive; SAH, severe alcoholic hepatitis; SAMe, S-adenosyl-l-methionine.

**Table 4:** Adverse events.

**Adverse events**	**Park et al., [** 18 **]**		**Higuera-De La Tijera et al., [** 21 **]**			**Thursz et al., [** 26 **]**			**Tkachenko et al., [** 22 **]**		**Tayek et al., [** 23 **]**		**Gawrieh et al., [** 20 **]**		**Szabo et al., [** 19 **]**		**Song et al., [** 24 **]**		**Pande et al., [** 25 **]**	
	**CST** **n** **(%)**	**PTX** **n** **(%)**	**CST** **n** **(%)**	**CST + MTD** **n** **(%)**	**PTX** **n** **(%)**	**CST** **n** **(%)**	**PTX** **n** **(%)**	**CST + PTX** **n** **(%)**	**CST** **n** **(%)**	**CST + SAMe** **n** **(%)**	**CST** **n** **(%)**	**CST + G-CSF** **n** **(%)**	**CST** **n** **(%)**	**A + Z** **n** **(%)**	**CST** **n** **(%)**	**A + PTX + zinc** **n** **(%)**	**CST** **n** **(%)**	**CST + rifaximin** **n** **(%)**	**CST** **n** **(%)**	**FMT** **n** **(%)**
SAEs	—	—	—	—	—	128 (47)	111 (41)	116 (42)	—	—	—	—	41 (56.2)	49 (66.2)	30 (60)	33 (62.3)	—	—	—	—
Infection	7 (11.9)	3 (4.9)	14 (40)	11 (31.4)	12 (36.4)	44 (24)	16 (11)	30 (19)	5 (25)	5 (30)	4 (22.2) 30 days 5 (27.7) 12 mg	3 (18.7) 30 days 4 (25) 12 mg	20 (27.4)	23 (31.1)	9 (18)	12 (22.6)	2 (6.9)	3 (14.2)	16 (28.7)	10 (18.2)
GI disorder	—	—	—	—	—	49 (27)	56 (39)	48 (30)	—	—	—	—	—	—	4 (8)	4 (7.5)	—	—	—	—
Gastrointestinal bleeding/variceal bleeding	5 (8.5%)	10 (16.4)	13 (37.1)	10 (28.6)	14 (42.4)	—	—	—	—	—	—	—	—	—	4 (8)	4 (7)	1 (3.4)	1 (4.8)	3 (5.3)	2 (3.6)
Hepatorenal syndrome	8 (13.6)	8 (13.1)	19 (54.3)	10 (28.6)	17 (51.5)	—	—	—	4 (20)	0	2 (11.1) 30 days 2 (11.1) 12 weeks	2 (12.5) 30 days 2 (12.5) 12 mg	—	—	—	—	2 (6.9)	0	—	—
Hepatic encephalopathy	7 (11.9)	5 (8.1)	21 (60.0)	10 (28.6)	17 (51.5)	—	—	—	—	—	0	0	—	—	—	—	5 (17.2)	1 (4.8)	—	—
Hepatobiliary disorders	—	—	—	—	—	27 (15)	24 (17)	23 (14)	—	—	—	—	—	—	3 (6)	9 (17)	—	—	—	—
AKI	—	—		—	—	—	—	—	—	—	2 (11.1) 30 days 3 (16.6) 12 mg	4 (25) 30 days 4 (25) 12 mg	16 (21.9)	33 (44.6)	13 (26)	10 (18.9)	—	—	2 (3.5)	1 (1.8)
Ascites	—	—	—	—	—	—	—	—	—	—	—	—	—	—	—	—	3 (10.3)	3 (14.2)	—	—
Renal and urinary disorders	—	—	—	—	—	10 (5)	13 (9)	8 (5)	—	—	—	—	—	—	15 (30)	13 (24.5)	—	—	—	—
Abdominal pain	8 (13.6)	6 (9.7)	—	—	—	—	—	—	—	—	—	—	—	—	—	—	—	—	—	—
Pneumonia nosocomial	—	—	—	—	—	—	—	—	2 (10)	4 (20)	—	—	—	—	—	—	—	—	—	—
Hyperglycemia	—	—	—	—	—	—	—	—	7 (35)	6 (30)	—	—	—	—	—	—	—	—	—	—
Fungal infections	—	—	—	—	—	—	—	—	—	—	—	—	—	—	5 (10)	0 (0)	—	—	—	—
Vascular disorders	—	—	—	—	—	—	—	—	—	—	—	—	—	—	10 (22)	7 (17)	—	—	—	—

Abbreviations: A, anakinra; CST, corticosteroid; FMT, fecal microbiota transplantation; G-CSF, granulocyte colony-stimulating factor; MTD, metadoxine; PTX, pentoxifylline; SAMe, S-adenosyl-l-methionine; Z, zinc.

## Data Availability

Data sharing is not applicable to this article as no new data were created or analyzed in this study.
